# Inhibition of nonenzymatic depurination of nucleic acids by polycations

**DOI:** 10.1002/2211-5463.12308

**Published:** 2017-09-22

**Authors:** Ran An, Ping Dong, Makoto Komiyama, Xiaoming Pan, Xingguo Liang

**Affiliations:** ^1^ College of Food Science and Engineering Ocean University of China Qingdao China; ^2^ Laboratory for Marine Drugs and Bioproducts Qingdao National Laboratory for Marine Science and Technology China; ^3^ National Institute for Materials Science (NIMS) Tsukuba Japan

**Keywords:** chitosan, depurination, DNA protection, polycations, spermine

## Abstract

DNA base depurination is one of the most common forms of DNA damage *in vivo* and *in vitro*, and the suppression of depurination is very important for versatile applications of DNA in biotechnology and medicine. In this work, it was shown that the polycations chitosan (Cho) and spermine (Spm) strongly inhibit DNA depurination through the formation of polyion complexes with DNA molecules. The intramolecular electrostatic interaction of positively charged polycations with DNA efficiently suppresses the protonation of purine groups, which is the key step of depurination. Importantly, the optimal pH for Cho's inhibition of depurination is significantly different from that of Spm. Cho is very effective in the inhibition of depurination in highly acidic media (pH: 1.5–3), whereas Spm is found to suppress the chemical reaction near neutral pH, as well as in acidic solutions. This remarkable pH specificity of the two biorelevant polycations is attributed to the difference in the *pK*_a_ values of the amino groups. The relevance of our results with the biological roles of biogenic polycations is also discussed.

**Abbreviations**
dAMP: 2′‐deoxyadenosine 5′‐monophosphate
N/P ratio: the ratio of amino groups in the polycation to the phosphates in DNA
ODNs: deoxyoligonucleotides

Depurination is one of the most prevalent forms of DNA damage in which the *N*‐glycosidic bonds are cleaved to release the corresponding adenine or guanine from DNA. This chemical reaction is especially predominant under acidic conditions. The reaction rate under physiological conditions in cells is not trivial and non‐negligible 1, 2. Previous studies confirmed that the protonation of purines in DNA is primarily responsible for this reaction 3. Furthermore, it has been well accepted that depurination causes significant problems in biotechnology applications of DNA. For example, the efficient depurination that occurs in acidic gastric juice and some acidic organelles (such as lysosomes) restricts the application of nucleic acid drugs 4. Besides, apurinic sites formed in genomic DNA often induce replication errors and thus become potential sources of spontaneous mutation. In addition, the breakage of phosphodiester bonds, which is fatal in some cases if not repaired, occurs much more easily at apurinic sites as compared with normal DNA. Enzymatic and nonenzymatic synthesis of long deoxyoligonucleotides (ODNs) also suffers from this damage 5. Therefore, efficient and facile methods to suppress the depurination are of crucial importance for further developments of DNA science and the relevant technology. Previously, several chemical modifications of DNA, especially at the sugar backbones, were successfully attempted to obtain depurination‐resistant DNA drugs and to understand more about the nature of depurination *in vivo* 6, 7, 8. To our knowledge, however, noncovalent approaches to stabilize DNA in its intact form and protect it from depurination have not yet been very successful.

On the basis of our previous finding that the depurination was notably decelerated by metal salts at high concentrations 3, we deduced that polycations bearing larger positive charges may inhibit depurination with much higher efficiency. In order to inhibit DNA depurination noncovalently, accordingly, we employ here the possible electrostatic interaction provided by positively charged polymers and oligomers. We expected that the protonation of purines (a key step of depurination reaction) is suppressed by electrostatic repulsion against the positive charges of polycations. The inhibitory effect may be especially promoted, because the polycations form polyion complexes with negatively charged DNA and locate the positive charges near the reaction centers 9. The present approach is reminiscent of previous works on the protection of DNA from various outer stimuli by polycations [e.g., chitosan, oligoamines, and positively charged proteins]. Through polyion complex formation, for example, DNA was protected from chemical agents, enzymatic degradation, X‐ray radiation, and others 10, 11, 12, 13, 14, 15, 16, 17, 18, 19, 20.

In this work, we select chitosan (Cho) and spermine (Spm) as the inhibitory polycations for depurination. Cho is composed of β‐1,4‐linkage of glucosamine and is used in many biomedical and pharmaceutical applications 21. Spm is one type of polyamine (including putrescine, spermidine, and Spm), which are present in all living cells including prokaryotes and eukaryotes, and it can stabilize the helical structure of DNA 22, 23, 24. Here, the dependencies of inhibitory effects of Cho and Spm on pH, the ratio of amino groups in the polycation to the phosphates in DNA (N/P) value, and DNA sequence were quantitatively analyzed. We found that depurination was greatly suppressed by Cho and Spm. In addition, the optimal pH for the inhibition by these two biorelevant polycations is significantly different from each other, and we can select from the two polycations according to the experimental requirements. The origin of this remarkable specificity is interpreted in terms of kinetic analysis. Furthermore, relevance of the present results to the biological roles of Spm and other intracellular polycations (e.g., histone) is also discussed.

## Results and Discussion

### Choice of DNA substrate for unbiased analysis of depurination

In this study, a pool of 30‐nt‐long ODNs of randomized sequences (N30) was used as DNA substrate unless noted otherwise. This is to avoid the complexity of analysis based on the sequence dependence of inhibitory effects 3. The amount of each of the purines (adenine and guanine), released from N30 during a predetermined reaction time, was evaluated by HPLC, and the conversion of depurination was determined in terms of the released amount with respect to the total amount in N30 (details are described in the Materials and methods section).

### Chitosan as eminent inhibitor for DNA depurination at highly acidic pH

Depurination of nucleic acids vigorously occurs in highly acidic solutions. Accordingly, the inhibitors under these conditions are of primary importance especially for nucleic acid drugs or functional food in stomach and some *in vitro* applications. Cho is the only biorelevant macromolecular polycations. Its amino groups have apparent *pK*_a_ of 6.4–6.5 25 and thus are almost completely protonated below pH 4.5.

**Figure 1:**
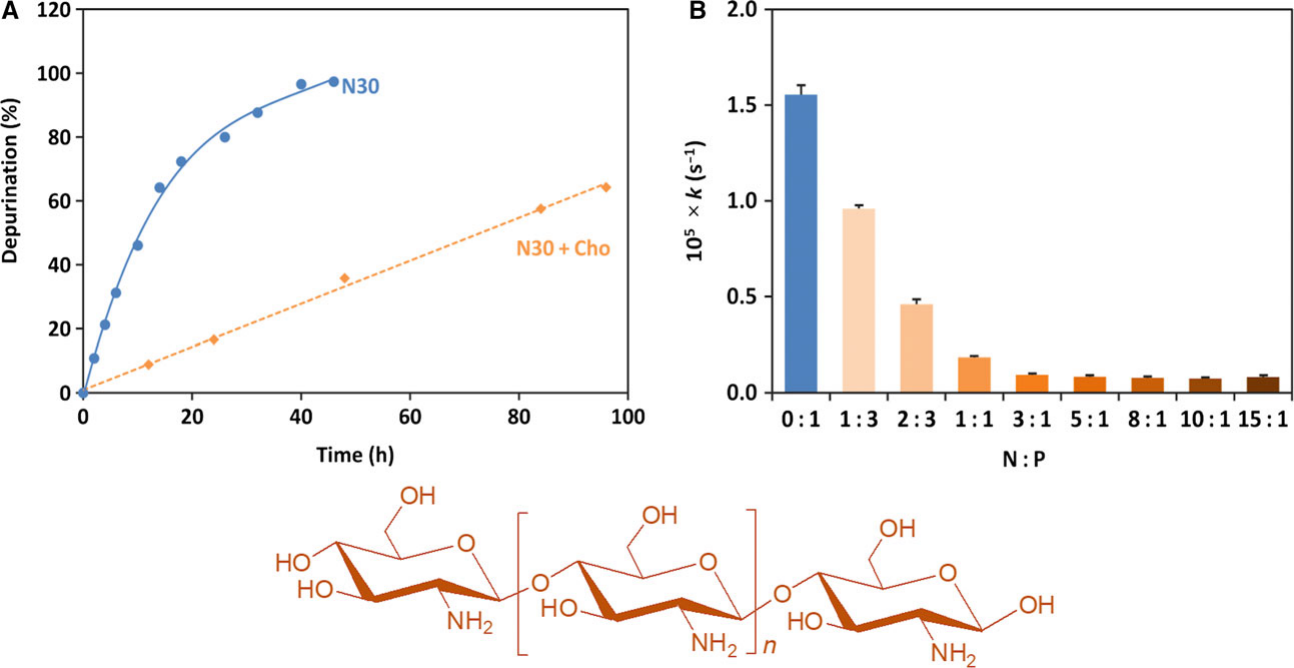
Inhibition by Cho of depurination of N30 at pH 2.0 and 37 °C. (A) Time courses in the presence of Cho (closed rhombuses) and its absence (closed circles); N/P = 3. (B) Effect of the amount of Cho on the first‐order rate constant of depurination. Structure of Cho is presented in the bottom panel.

Depurination from N30 substrate at pH 2.0 and 37 °C was significantly reduced by the addition of Cho with molecular weight of 6500 Da (Fig. 1A). In the presence of Cho (N/P = 3), for example, the conversion of depurination was < 36% after 48 h (the orange rhombuses). In the absence of Cho (blue circles), however, almost all the purine groups were removed from N30. The pseudo‐first‐order rate constants of depurination under these conditions are 9.4 × 10^−7^ and 1.6 × 10^−5^·s^−1^, respectively. As shown in Fig. 1B, the inhibitory effect monotonously increased with increasing amount of Cho and virtually reached a plateau at N/P = 3. This indicates that a polyion complex between negatively charged N30 and positively charged Cho is almost completely formed, when N/P ratio is 3 or larger.

**Figure 2:**
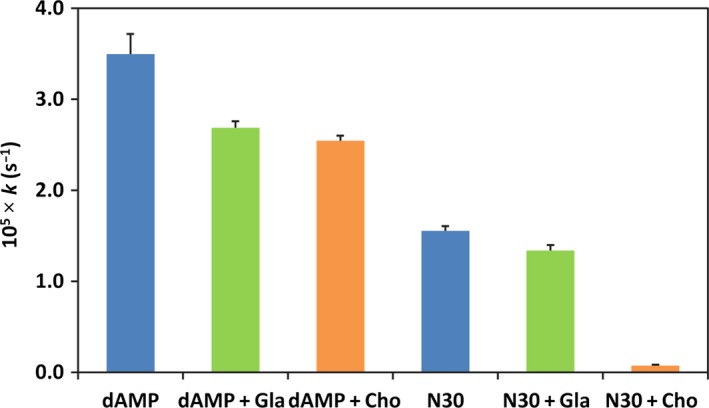
The inhibition by Cho and glucosamine (Gla) on depurination from dAMP, and the inhibition by glucosamine (Gla), in place of Cho, on the depurination of N30. N/P = 3 at pH 2.0 and 37 °C.

In contrast with this remarkable inhibitory effect of macromolecular Cho, its monomeric unit, glucosamine, showed only a marginal inhibition (compare the two rightmost bars in Fig. 2). Furthermore, Cho hardly inhibited the depurination of 2′‐deoxyadenosine 5′‐monophosphate (dAMP), a monomeric analog of a nucleotide in N30 (see the third bar from the left). The depurination rate from dAMP without glucosamine was almost the same as that in the presence of glucosamine (the second bar from the left). The data strongly suggested that the inhibition of depurination by Cho requires the formation of polyion complex between this polymeric cation and the DNA as polymeric anion.

**Figure 3:**
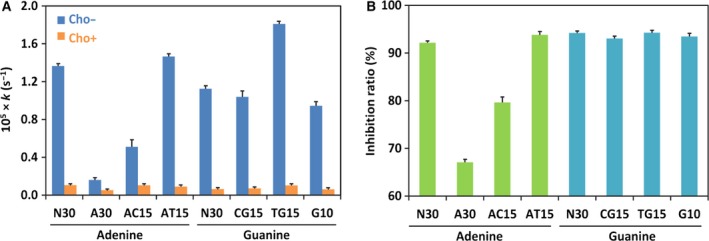
Inhibitory effect of Cho for the depurination of ODNs of various sequences. (A) First‐order rate constants for the release of each of adenine and guanine; (B) the corresponding inhibitory ratios. N/P = 3 at pH 2.0 and 37 °C. The sequences of ODNs are listed in Table 1.

The importance of electrostatic interactions between Cho and DNA substrate for the inhibition was further substantiated by the remarkable effect of DNA sequence on the inhibition. In this experiment, several repetitive sequences (Table 1) were used to amplify the sequence effects. As shown in Fig. 3A, the depurination of A30 and AC15 was suppressed by Cho at pH 2.0 in considerably smaller magnitude than the depurination of other ODNs. It should be noted that these ODNs are composed of only adenine and cytosine, which are almost completely protonated in the reaction media (their *pK*_a_ values are around 4). Thus, the negative charges of phosphate backbones of these ODNs are fully compensated by the positive charges on the DNA bases, being highly unfavorable to form polyion complexes with positively charged Cho 3. In order to facilitate the comparison of the magnitude of inhibition of Cho, the inhibitory ratio (*I*_r_) was defined by [(*k*_0_ − *k*_p_)/*k*_0_] × 100 (%). Here, *k*_p_ and *k*_0_ are the first‐order rate constants of depurination in the presence and the absence of Cho, respectively. As expected, the *I*_r_ values for A30 and AC15 were much smaller than the values for the other sequences (see Fig. 3B). In the absence of Cho, for the similar reason, these two ODNs are depurinated more slowly than the others, because the protonated adenine or cytosine suppressed the depurination. The inhibitory effect by Cho was the highest for AT15 or TG15, because thymine is not protonated even under highly acidic conditions.

**Table 1:** DNA sequences used for depurination

Name	Sequence (5′→3′)
N30	NNNNN NNNNN NNNNN NNNNN NNNNN NNNNN
A30	AAAAA AAAAA AAAAA AAAAA AAAAA AAAAA
AC15	ACACA CACAC ACACA CACAC ACACA CACAC
AT15	ATATA TATAT ATATA TATAT ATATA TATAT
TG15	TGTGT GTGTG TGTGT GTGTG TGTGT GTGTG
CG15	CGCGC GCGCG CGCGC GCGCG CGCGC GCGCG
G10	GGGGG GGGGG

**Figure 4:**
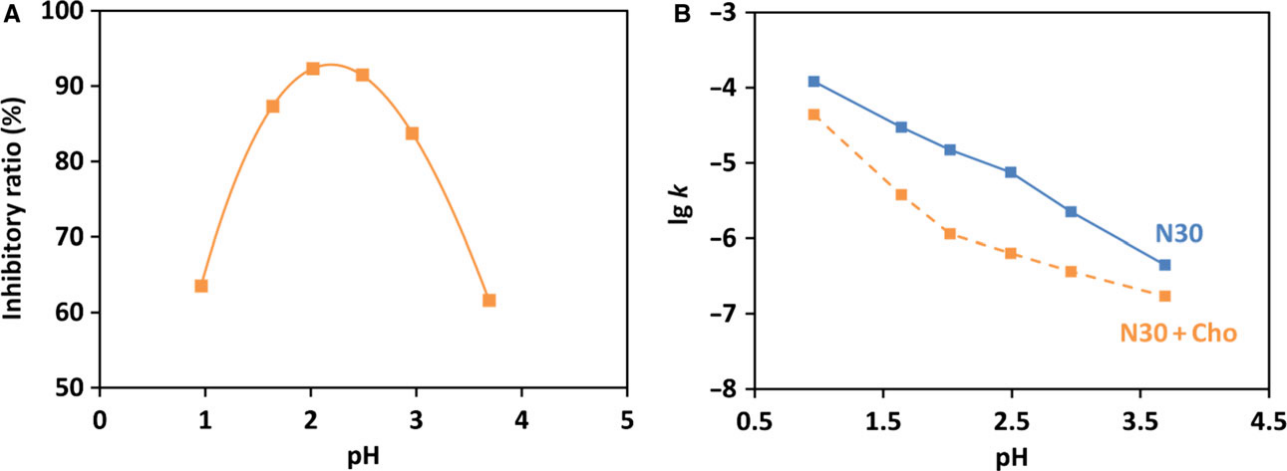
Inhibitory effect of Cho on depurination under various pH. (A) pH dependence of the inhibitory ratio *I*_r_ of Cho for the depurination of N30. (B) The rate constants of depurination in the presence and absence of Cho under various pH. N/P = 3 at 37 °C.

In Fig. 4A, the inhibitory ratio (*I*_r_) of Cho on the depurination of N30 was plotted as a function of pH. The *I*_r_ value showed a bell‐shaped profile with the maximum at around pH 2, where the depurination rate of N30 was decelerated to only 8% of the intrinsic reaction rate (*k*_0_). The increase in *I*_r_ with decreasing pH in the right‐hand part of the bell shape is primarily ascribed to the suppression of protonation of adenine at N‐7 (*pK*_a_ = 4.15) by the positive charges of Cho. The second protonation (at the N‐3 position), which further facilitates the depurination 3, 26, should be also suppressed by Cho. On the other hand, the left‐hand part of the bell shape is mainly due to gradual protonation of phosphate groups (*pK*_a_ = 1.5) in the DNA backbone with decreasing pH. The guanine groups (*pK*_a_ = 3.20) in N30 substrate should be also gradually protonated. Under these conditions, the net charges of DNA are only slightly negative (or even slightly positive) and cannot form a stable polyion complex with Cho. It is noteworthy that the protonation of adenine and cytosine at around pH 4 imposed no drastic effect on the rate constant *k*_p_ for the depurination in the presence of Cho (Fig. 4B). Apparently, the polyion complex between Cho and N30 was kept intact even when these adenine groups were protonated (but it was not the case when the guanines were also protonated at a still lower pH; *vide ante*). As described in the following section, the complex between Spm and N30 was significantly less stable and more susceptible to the protonations of DNA.

### Inhibitory effects of the combinations of Cho with metal ions

**Figure 5:**
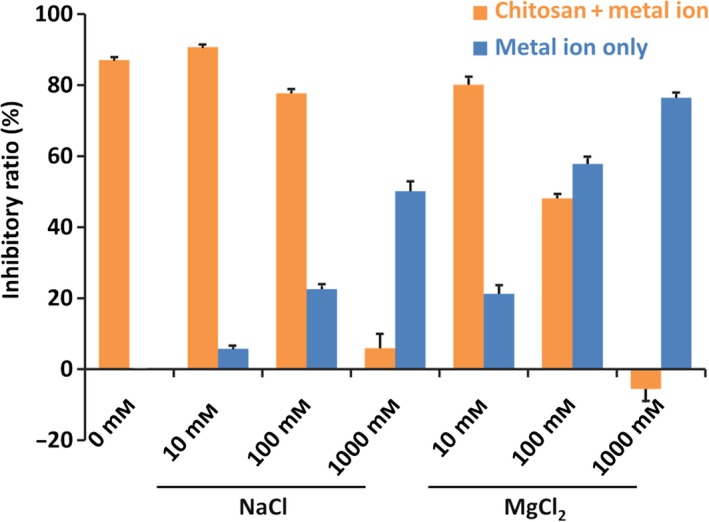
Inhibitory effect of Cho on the depurination of N30 in the presence of metal ions (orange bars). The blue bars are the inhibitory effect of metal ions only. N/P = 3 at pH 1.4 (HCl) and 37 °C.

In previous studies, high concentrations of Na^+^ (> 100 mm) and Mg^2+^ (> 10 mm) were found to suppress the depurination of DNA 3, 27. On the other hand, the inhibitory effect by Cho was significant even at a concentration of lower than 9 μm (N/P = 1). As the inhibitor of depurination, Cho is more than 1000 times more efficient than these metal ions. We were interested to find out how the activity of Cho was affected in the presence of metal ions. We investigated the inhibitory effect of combination of Cho and metal ions (the orange bars in Fig. 5). For the purpose of comparison, inhibitory effects of the metal ions alone (in the absence of Cho) were also shown (the blue bars). Significantly, the inhibitory effect of Cho was notably deteriorated by coexisting Na^+^ (> 100 mm) and Mg^2+^ (> 10 mm). With 1000 mm Na^+^ or Mg^2+^, the Cho had almost no inhibitory effect on depurination (the combination of Cho with 1000 mm Mg^2+^ slightly accelerated the reaction, as shown by ‘negative’ *I*_r_ value). Competitive binding of the Cho and the metal ions to the N30 was evident. In other words, the suppression of Cho on depurination should have the same innate character as the suppression by the metal ions. The results in Fig. 5 also indicate that Cho can satisfactorily inhibit the depurination even in *in vivo* applications. The metal ion concentrations in cells are not very high ([K^+^] = 140 mm, [Na^+^] = 10 mm, and [Mg^2+^] = 0.5 mm) and thus may not counteract the inhibition activity of Cho.

### Efficient inhibition of DNA depurination at around pH 7 by Spm

As shown above, Cho is a very effective inhibitor of depurination of ODNs at highly acidic pH. However, the effect is not so remarkable near neutral pH, as its amino groups (*pK*_a_ = 6.4–6.5 25) are not fully protonated under these conditions and thus the formation of polyion complexes with ODN substrates is not very efficient. In order to obtain an inhibitor that functions still more effectively under physiological conditions, polyamines were employed. It was reported that polyamines including putrescine, spermidine, and Spm protected DNA against depurination at pH 4.3, and among these polyamines, Spm was the most efficient protector 20. Spm, a biogenic tetraamine, has *pK*_a_ values for stepwise protonation of 10.80, 10.02, 8.85, and 7.96. Thus, most of the primary and the secondary amino groups are protonated at around pH 7, allowing this tetraamine to form multi‐ion complex with DNA and protect it in a wide range of pH.

**Figure 6:**
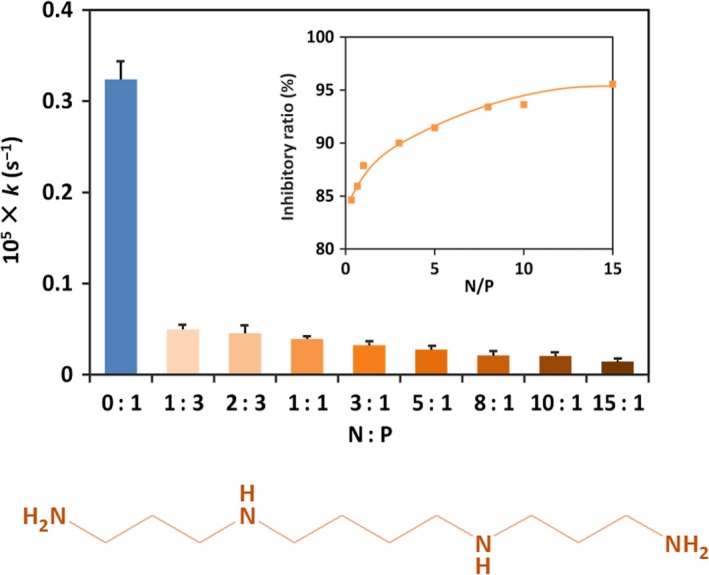
Effect of Spm concentration on the rate of depurination from N30 at pH 3.0 and 37 °C. The inserted figure shows the inhibitory ratios. Structure of Spm is presented in the bottom panel.

**Figure 7:**
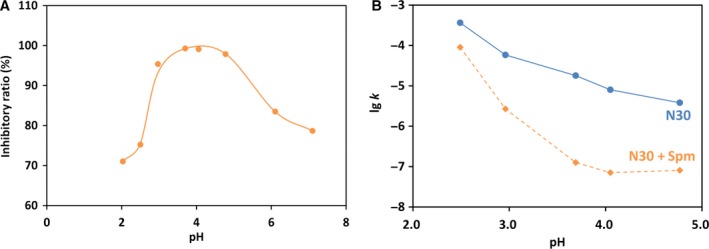
Inhibitory effect of Spm on depurination under various pH. (A) pH dependence of the inhibitory ratio (*I*_r_) of Spm for the depurination of N30 (N/P = 10). (B) The rate constants of depurination in the presence and absence of Spm under various pH. The *I*_r_ value at pH 2.5–4.8 was measured at 60 °C, whereas the value at pH 6.0–7.0 was at 37 °C, simply because of experimental convenience (note that the *I*_r_ value was kept almost unchanged between 35 °C and 60 °C). In (B), the pH profile of each of *k*_p_ and *k*_0_ was presented where all the values were measured at 60 °C.

As shown in Fig. 6, Spm remarkably inhibited the depurination of N30. The inhibitory effect increased with increasing N/P, as expected. At the optimal pH (pH = 3.7), the depurination was almost completely inhibited by Spm when N/P = 10 (*I*_r_ = 99%; see Fig. 7A).

Interestingly and most importantly, the inhibition by Spm is very effective in a wide range of pH covering from pH 7 down to pH 2. At pH 7, for example, its inhibition is very strong and the *I*_r_ value is close to 80%. Important roles of Spm in biological functions are strongly indicated.

It is noteworthy that *I*_r_ gradually becomes smaller when the pH is decreased below 4. This is quite significant in contrast with the inhibition by Cho, in which *I*_r_ monotonously increases with decreasing pH in this pH range (compare Fig. 7A with Fig. 4A). At around pH 4, the adenine and cytosine groups in N30 start to be protonated, and the inhibitory effect of Spm is largely deteriorated by these protonations. Consistently, the rate constant (*k*_p_) of the depurination in the presence of Spm (the orange line in Fig. 7B) is almost constant down to pH 4, and steeply increases below pH 4 (note that the logarithm of *k*_p_ is plotted here). This curve can be fairly interpreted in terms of the following mechanism: (a) The polyion complex between Spm and N30 is not very stable and decomposed when both the adenine and cytosine groups in N30 are protonated and (b) the N30, which is removed from the polyion complex due to its decomposition, is subjected to conventional proton‐catalyzed depurination. Both of these two factors should drastically increase *k*_p_ with decreasing pH. Accordingly, the slope of the log *k*_p_ vs. pH plot in pH 2.5–3.7 is very steep and close to −2. On the other hand, *k*_0_ in the absence of Spm (the blue line in Fig. 7B) is fundamentally proton‐catalyzed reaction, and thus, the slope of log *k*_0_ vs. pH plot is around −1. Detailed analysis of the mechanism of the latter depurination (the intrinsic reaction in the absence of polycations) was presented previously 3.

### Protection of genomic DNA from depurination by polycations

Both Cho and Spm notably inhibited the depurination of single‐stranded and double‐stranded genomic DNAs from salmon (salmon ssDNA and salmon dsDNA in Table 2). The rate constant of depurination of salmon dsDNA at pH 5.1 and 60 °C in the presence of Cho (N/P = 3) was 6.8 × 10^−9^ s^−1^, while the value with Spm (N/P = 10) was 5.4 × 10^−9^ s^−1^. These values correspond to the suppression of depurination by 30‐ to 40‐fold. It is noteworthy that Cho efficiently protected the genomic DNA although the reaction pH (pH = 5.1) was considerably higher than its optimal value for the protection of N30 (Fig. 4A). Because of macromolecular polymer effect, the large DNA molecules can efficiently form a polyion complex with Cho, even when the amino groups of the Cho are only partially protonated.

**Table 2:** Rate constants (*k*, s^−1^) of depurination from genomic DNA in the presence of Cho and Spm. For Cho, the N/P value was 3, and for Spm, the N/P value was 10. Depurination of genomic DNA at pH 5.1 was performed under 60 °C, and depurination at pH 7.1 and pH 2.0 was performed under 37 °C

	Salmon ssDNA	Salmon dsDNA		
	pH 5.1	pH 5.1	pH 7.1	pH 2.0
DNA	4.5 × 10^−7^	1.9 × 10^−7^	4.5 × 10^−11^	1.6 × 10^−5^
DNA + Cho	4.5 × 10^−8^	6.8 × 10^−9^	–	1.3 × 10^−6^
DNA + Spm	1.0 × 10^−8^	5.4 × 10^−9^	1.7 × 10^−11^	–

Significantly, Spm showed notable protection of salmon dsDNA under physiological conditions (pH 7.1 and 37 °C). The protection was about threefold when N/P = 10. Here, the reaction rate was estimated after 120 days, as the depurination was extremely slow under these conditions. Spm is commonly present in nuclei 19, and the positively charged histones are wrapping around genomic DNA in chromatin. Thus, our results are likely relevant to the maintenance of integrity and stability of genomic DNA *in vivo*.

## Conclusion

It has been found that polycations significantly suppress DNA depurination under acidic and physiological conditions. Cho is very effective in the inhibition of depurination in highly acidic media (pH: 1.5–3), whereas Spm is able to suppress the reaction in a wide pH range (pH: 3–7). The protection is ascribed to the electrostatic interaction in the polyion complexes with DNA. Thus, a number of cations should be located at appropriate intervals, and the protection activity is straightforwardly determined by acid–base properties of the amino residues.

The strong inhibition effect and rather simple inhibition method of depurination make polycations being easily extended to various applications. For example, the stability of DNA fragments in acidic conditions can be promoted by polycations and the transfection of these fragments into cells through endocytosis is facilitated, resulting in still more efficient therapies by antigene and/or antisense effects, siRNA strategy, etc. Alternatively, for indigested nucleic acid medicine or functional foods containing DNA, the addition of Cho may protect these nucleic acids from depurination under strong acidity in our stomach. This may be helpful for understanding the effect of Cho as the functional components in dietary supplements.

## Materials and methods

### Materials

Low molecular weight Cho (6500 Da; Haili, Yantai, China), Spm (Sigma‐Aldrich, Milwaukee, WI, USA), and glucosamine (Sigma‐Aldrich) were obtained from commercial sources. In this Cho specimen, 91.1% of the monomeric units are glucosamine and bear an amino group (the residual 8.9% has an acetamide group, in place of amino group, due to incomplete hydrolysis of chitin as the starting resources). The oligonucleotides (ODNs) were ordered from Integrated DNA Technologies, Inc. (Coralville, IA, USA). dAMP, salmon sperm double‐stranded DNA, and salmon single‐stranded DNA were purchased from Sigma‐Aldrich (St. Louis, MO, USA). Lambda DNA was purchased from New England Biolabs, Inc (Ipswich, MA, USA).

### Depurination of DNA

Typical reaction mixture consists of DNA substrate, polycation, and uracil as the internal standard for HPLC analysis in 50 mm phosphate buffer of various pH values. The final concentrations of substrate were 10 μm (for ODNs), 300 μm (for dAMP), and 30 μg·mL^−1^ (for salmon dsDNA or salmon ssDNA). In control experiments, sterilized water was used in place of the solutions of polycations.

Depurination reaction was performed at a constant temperature on a TC‐5000 thermal cycler (Techne, Staffordshire, UK) for several hours or days. To quench the reaction, pH values of aliquots were adjusted to 7.0–8.0 by adding 10 μL NaOH solution of appropriate concentration.

### HPLC analysis of reaction products

After a predetermined time, the products of depurination reactions were directly analyzed with an HPLC system (Elite, Dalian, China) using a YMC C18 column (250 × 4.6 mm, particle size 5 μm; YMC, Kyoto, Japan). The amounts of adenine and guanine, released from the DNA, were quantitatively measured. Independently, the DNA substrate was treated under strong conditions to complete the depurination, and the total amount of purines in the DNA was determined by HPLC. The percentage of depurination was evaluated as described in our previous paper 3, and the average value for depuration of adenine and guanine was used unless noted otherwise. In the reactions of salmon DNA, the reaction products were first treated with 1 U DNase I (Thermo Scientific, Pittsburgh, PA, USA) at 37 °C for 10 min to digest the macromolecular DNA, and then subjected to HPLC analysis.

## Author contributions

RA, XL, and PD planned experiments; RA and XP performed experiments; RA and KM analyzed data; RA and PD contributed reagents or other essential materials; RA, XL, and KM wrote the manuscript.
